# P053: Secular trends of methicillin-resistant Staphylococcus aureus (MRSA) at Geneva University Hospitals (HUG) over a 12-year period

**DOI:** 10.1186/2047-2994-2-S1-P53

**Published:** 2013-06-20

**Authors:** C Fankhauser, J Schrenzel, P Francois, D Pittet, S Harbarth

**Affiliations:** 1 Infection Control Program, Geneva University Hospitals, Geneva, Switzerland; 2 Bacteriology Laboratory, Geneva University Hospitals, Geneva, Switzerland; 3 Genomic Research Laboratory, Geneva University Hospitals, Geneva, Switzerland

## Introduction

In 2000, the introduction of the highly epidemic ST228 South-German MRSA clone at HUG coincided with a progressive increase in MRSA burden.

## Objectives

To describe secular trends of MRSA rates at HUG, related to infection control measures.

## Methods

In 1993 initiated a multifaceted MRSA prevention program, including patient screening, decontamination, surveillance, contact isolation, a computerized alert system and a hospital-wide hand hygiene (HH) promotion campaign. Since 2003, it was strengthened by an educational campaign of all personnel; 2005, by routine MRSA genotyping of SCCmec elements; and 2006 by a 2^nd^ HH campaign with periodic audits and feedback. Universal screening on admission, discharge and weekly was only performed in the intensive care unit since 2004. MRSA surveillance included: (1) incidence rates of hospital acquired (HA)-MRSA infection or colonization; (2) HA-MRSA bloodstream infections (BSI); (3) the proportion of MRSA among *S. aureus* BSI; (4) incidence rates of MRSA- clinical cultures (CC).

## Results

At HUG, from 2000-2012, 12347 patients were documented as MRSA-colonized or infected (incl. >75% screening swabs; 507 BSI episodes); 8331 were considered HA-MRSA. As from 2000, annual rates of all indicators showed an increasing trend, and declined in the last few years. New HA-MRSA cases per 100 admissions increased from 1.36 to 2.00 (2006) and declined to 0.79 (2012). Incidence density of cases per 1000 hospital-days showed the following trends: HA-MRSA, from 0.92 to 1.36 (2007) to 0.55 (2012); ICU-acquired HA-MRSA from 2.3 (2002) to 10.5 (2006) to 2.39 (2012); MRSA-positive CC rates from 0.68 to 1.44 (2008), to 0.41 (2012); HA-BSI from 0.049 to 0.07 (2009), to 0.016 (2012). The proportion of MRSA among *S. aureus* BSI remained over 34% for 10 years, declined to 20% in 2012. The predominance of MRSA containing SCCmecI decreased from 83% in 2005 to 64% in 2012.

## Conclusion

MRSA rates have decreased in the last four years.An ongoing multifaceted prevention program helped to contain endemic MRSA rates. The decay of MRSA’s predominant clone might also have influenced this decrease.

## Disclosure of interest

None declared

